# Depression and anxiety in cancer patient enrolled in clinical trials with serious adverse events

**DOI:** 10.1002/cam4.6556

**Published:** 2023-09-18

**Authors:** Zhen Peng, Chongwei Wang, Yubei Sun, Yan Ma, Jumei Wang, Fei Xu, Xiaoling Xu, Yin Chen

**Affiliations:** ^1^ Division of Life Sciences and Medicine, Drug Clinical Trial Institution, The First Affiliated Hospital of USTC University of Science and Technology of China Anhui Hefei China; ^2^ Division of Life Sciences and Medicine, Department of Oncology, The First Affiliated Hospital of USTC University of Science and Technology of China Hefei Anhui China; ^3^ Division of Life Sciences and Medicine, Department of Rheumatology and Immunology, The First Affiliated Hospital of USTC University of Science and Technology of China Hefei Anhui China; ^4^ Division of Life Sciences and Medicine, Department of Endocrinology, The First Affiliated Hospital of USTC University of Science and Technology of China Hefei Anhui China; ^5^ Division of Life Sciences and Medicine, Department of Pulmonary and Critical Care Medicine, The First Affiliated Hospital of USTC University of Science and Technology of China Hefei Anhui China; ^6^ Division of Life Sciences and Medicine, Department of Scientific Research, The First Affiliated Hospital of USTC University of Science and Technology of China Hefei Anhui China

**Keywords:** anxiety, cancer, clinical trial, depression, SAE

## Abstract

**Objective:**

Drug‐induced side effects, particularly serious adverse events (SAEs), often affect cancer patients enrolled in clinical trials. However, little is known about anxiety and depression in cancer patients who experienced SAEs. This study evaluated the prevalence of anxiety and depression in cancer patients enrolled in clinical trials who experienced SAEs and explored the risk factors.

**Methods:**

A multi‐center, cross‐sectional survey was conducted in hospitals affiliated with the University of Science and Technology of China from December 2021 to November 2022. A total of 112 cancer patients who experienced SAEs while enrolled in clinical trials, and who completed the informed consent process and study questionnaires, were included in the final analysis.

**Results:**

The rate of moderate–severe depression in cancer patients was 38.4% and that of moderate–severe anxiety was 13.4%. Among the patients who had moderate–severe anxiety, 93.3% had concurrent moderate–severe depression. Lower cognitive function and lower global quality of life were risk factors for depression in cancer patients who experienced SAEs. Pain, low emotional function, low global quality of life, and a high Impact of Events Scale score were risk factors for anxiety.

**Conclusions:**

Cancer patients enrolled in a clinical trial who experienced SAEs tended to be anxious and depressed, particularly the latter. These results indicate the need to evaluate anxiety and depression, and mental health treatment among cancer patients with SAEs in clinical trials.

## 1 BACKGROUND

Cancer patients enrolled in clinical trials often experience physical and mental symptoms.^1^ The most common mental health disorder symptoms are anxiety and depression. Studies have shown that 14% and 12% of cancer patients experience moderate–severe symptoms of anxiety and depression. Depression was also reported in a review of phase I clinical trials.^2^ In addition, many participants in clinical trials have a relatively poor understanding of the process, which can impose a psychological burden after the occurrence of serious adverse events (SAEs),^3^ defined as an adverse medical event that occurs in response to any dose of a drug, including death, life threatening illness, inpatient hospitalization or prolonged hospitalization, persistent or significant disability/incapacity, and congenital anomalies/birth defects.^4^

In clinical trials, SAEs are an important measure of drug safety and they can lead to discontinuation of treatment, hospitalization, or death. In addition to being physically painful for cancer patients participating in clinical trials, SAEs impose a psychological burden that may result in anxiety or depression.^5^ Cancer patients are increasingly being recommended to take part in clinical trials, as the number of clinical studies of cancer has risen sharply in recent years.^6^, ^7^ However, treatment uncertainties and drug‐induced side effects, particularly SAEs, may affect cancer patients.^8^ According to some reports, about 200,000 SAEs were reported in China in 2021, mainly in relation to new anti‐cancer drugs.^9^ In one study, 25% of patients did not participate in clinical trials because of concerns about SAEs and,^10^ in another SAEs accounted for 27.06% of cancer patient dropout from clinical trials.^8^

Anxiety and depression subsequent to SAEs decrease the quality of life of cancer patients, which, for those enrolled in clinical trials, can lead to poor adherence and,^11^ in turn, exacerbate anxiety about adverse treatment reactions.^12^ Rehabilitation and survival are also affected by anxiety and depression, which may prolong hospitalization.^13^ According to the findings of a meta‐analysis, patients with depression had a 17% higher relative risk of dying than those without depression.^14^ Another study similarly reported that depression is associated with poorer survival.^15^

Emotional distress is a key component of cancer treatment in clinical trials. The American Society of Clinical Oncology (ASCO) has published guidelines for screening and treating depression and anxiety symptoms in cancer patients and has identified risk factors for depressive and anxiety symptoms including sex, marital status, and age, among others.^16^ The incidence rates of anxiety and depression in women are often higher than in men, and the prevalence of anxiety and depression is higher in younger than older groups.^17^, ^18^ Single, divorced, and widowed cancer patients may lack emotional support, leading to increased susceptibility to anxiety and depression.^19^ A history of mental illness, alcohol or drug abuse, recurrent, advanced, or progressive disease, low socioeconomic status, and other chronic medical conditions affect anxiety and depression in cancer patients.^20^ In addition, poorer quality of life, less social support, and more stressful life events during the past year are associated with depressive symptoms in cancer patients.^21^

Psychological evaluation is not routinely conducted in cancer clinical trials in China. Reports of anxiety and depression among patients in our center were brought to our attention, where these conditions can affect clinical trials and the rehabilitation of patients.^2^ Although many studies have been performed on anxiety and depression in cancer patients, few have been concerned with anxiety and depression in cancer patients who have experienced SAEs, and the current prevalence of anxiety and depression in this population is unclear.^13^ Therefore, this study evaluated anxiety and depression in cancer patients enrolled in clinical trials who experienced SAEs, and explored the risk factors to improve understanding of these conditions in this population, and identify those at higher risk, to aid early detection and timely treatment.

## 2 METHODS

### 2.1 Study design and study population

A multi‐center, cross‐sectional survey was conducted in three hospitals affiliated with the University of Science and Technology of China from December 2021 to November 2022. The participants were cancer patients enrolled in oncology clinical trials who had experienced SAEs, were aged ≥18 years, were able to understand and complete the questionnaires independently, and were able to provide informed consent. The exclusion criteria were depression/anxiety or a history of depression/anxiety when enrolled in a clinical trial, neurological problems, refusal to consent, and cognitive impairment of any etiology. A total of 112 cancer patients who had experienced SAEs and completed the informed consent process and questionnaires were included in the final analysis.

### 2.2 Procedure

SAEs among the cancer patients were promptly treated following their occurrence. Between 3 and 7 days later, the researchers obtained demographic information after screening and informed consent, and assisted the participants with respect to the questionnaires. The data collectors in all research centers were trained in a standardized manner, including in terms of the research protocol, questionnaire, and communication skills. The meaning of each question was explained to the participants in detail. The questionnaires were sent to the participants, who completed and returned them. The whole process of receiving, completing, and returning the questionnaires was confidential.

The demographic information collected included sex, age, marital status, occupation, education level, living conditions, frequency of acquisition of medical information, whether the individual was participating in a clinical trial for the first time, type of cancer, and other characteristics. Patients were provided with study questionnaires including the Charlson Comorbidity Index (CCI), which is a 19‐item instrument that measures various chronic illnesses and has a total score of 1–6 (depending on the mortality risk).^22^ This questionnaire has been validated as a predictor of health outcomes in cancer patients.^23^ The CCI had a Cronbach's alpha of 0.897 in this study. The Impact of Events Scale (IES) is a 22‐item instrument that assesses reactions to a wide range of stressors.^24^ Higher scores reflect more cancer‐specific stress. The reliability of the Chinese version of the IES has been verified.^25^ The IES had a Cronbach's alpha of 0.974 in this study. Life events is a 5‐item measure that assesses major negative stressors in the past year.^26^ The Life events scale had a Cronbach's alpha of 0.691 in this study. The fatigue symptom inventory (FSI) is an 11‐item instrument used to evaluate signs of fatigue.^27^ The reliability of the Chinese version of the FSI has been verified.^28^ The FSI had a Cronbach's alpha of 0.975 in this study. The European Organization for Research and Treatment of Cancer (EORTC) QLQC30 (ver. 3) was developed to assess the quality of life of cancer patients.^29^ The QLQC30 comprises nine multi‐item scales including five functional scales (Physical, Role, Cognitive, Emotional, and Social), three symptom scales (Fatigue, Pain, and Nausea and vomiting), and a global health and quality of life scale.^30^ Higher scores indicate better functioning. Cronbach's alpha (0.956) was calculated to assess the reliability. A pain intensity numerical rating scale ranging from 0 to 10 (0 = no pain and 10 = the worst pain imaginable) was used to assess pain.^31^

The main indicators of depression and anxiety were the PHQ‐9 and GAD‐7 scores. The PHQ‐9 is a nine‐item measure of symptoms of depression.^32^ Item scores are summed; total scores range from 0 to 27. Symptoms are classified on the basis of the PHQ‐9, as follows: none‐mild, score of 1–7; moderate, score of 8–14; moderate‐to‐severe, score of 15–19; and severe, score of 20–27.^16^ The PHQ‐9 had a Cronbach's alpha of 0.934 in this study. The GAD‐7 is a 7‐item measure of symptoms of anxiety, which are classified as follows: none‐mild, score of 0–9; moderate, score of 10–14; and moderate‐to‐severe, score of 15–21.^16^ The GAD‐7 had a Cronbach's alpha of 0.960 in this study. The none‐mild group included none‐mild, and the moderate–severe group included moderate, moderate‐to‐severe, and severe.

### 2.3 Statistics

Descriptive statistics for sex, marital status, income, life events, fatigue, cancer type, depression, and anxiety were generated using SPSS 24 software (SPSS Inc.), which was also used for subsequent analyses. Normality was evaluated in a Kolmogorov–Smirnov test. Demographic characteristics were summarized as a percentage for categorical variables and as the median and inter‐quartile range (IQR) for non‐parametric variables. Demographic characteristics and the CCI, IES, life events, FSI, EORTC QLQ‐C30, and pain intensity numerical rating scale scores were analyzed using a Mann–Whitney *U*‐test/Kruskal–Wallis *H*‐test for non‐parametric variables and Fisher's exact/chi‐squared test for categorical variables. Spearman's correlation analysis was performed to assess the relationships between EORTC QLQ‐C30 and the PHQ‐9/GAD‐7. Associated factors were explored using logistic regression analysis, and were expressed as odds ratios (OR) with 95% confidence intervals (CIs). Multivariate models were constructed using the stepwise selection technique including all potential variables such as demographic characteristics and previously supported risk factors. The ORs and two‐sided P‐values were calculated. A *p*‐value <0.05 was considered significant for all tests.

## 3 RESULTS

Table 1 showed the demographic characteristics of the participants. A total of 122 cancer patients were screened and 112 were included in the final analysis. Among these patients, eight did not complete the questionnaire and two withdrew informed consent. A total of 56 males (50%) and 56 females (50%) were included, with a median age of 59 years; 108 (96.4%) were married, 53 (47.3%) were unemployed, 38 (33.9%) were retired, 92 (82.1%) had a high school education or below; 19 (17%) lived alone, 44 (39.3%) were receiving treatment for the first time, 104 were participating in a clinical trial for the first time (92.9%), and 76 (67.9%) were enrolled in a phase III clinical trial. The main types of cancer were lung cancer (*n* = 23 [20.5%]), breast cancer (*n* = 25 [22.3%]), liver cancer (*n* = 19 [17.0%]), and gastric cancer (*n* = 18 [16.1%]). The other cancer types are shown in Table 1.

**TABLE 1:** Participant demographic characteristics (*N* = 112).

Variables	Number	Percentage
Sex		
Male	56	50.0
Female	56	50.0
Age (years), median (inter‐quartile range)	59.0 (12.0–66.8)	
Marital status		
Married	108	96.4
Unmarried	4	3.6
Occupation		
Full‐time	13	11.6
Part‐time	3	2.7
Unemployed	53	47.3
Retired	38	33.9
Others	5	4.5
Education level		
High school level or below	92	82.1
College/university and above	20	17.9
Living alone		
Yes	19	17.0
No	93	83.0
Frequency of acquiring medical information		
Frequently (several times per month)	89	79.5
Occasionally (several times per year)	23	20.5
First treatment		
Yes	44	39.3
No	68	60.7
Participating in clinical trials for the first time		
Yes	104	92.9
No	8	7.1
Stage of clinical trial		
I	15	13.4
II	21	18.8
III	76	67.9
Type of cancer		
Lung	23	20.5
Breast	25	22.3
Liver	19	17.0
Gastric	18	16.1
Esophagus	3	2.7
Cervical	4	3.6
Ovarian	3	2.7
Prostate	3	2.7
Hematologic	7	6.3
Other	7	6.3

Table 2 lists the factors influencing depression and anxiety in cancer patients who experienced SAEs. None‐mild depression was determined in 69 (61.6%) participants and moderate–severe depression in 43 (38.4%). The median PHQ‐9 score of the none‐mild and the moderate–severe depression groups were 2 (0–4) and 10 (8–14), respectively. The rate of frequent acquisition of medical information (*n* = 39 [90.7%]) was higher in the moderate–severe group than in the none‐mild group (50 [72.5%]) (*p* = 0.020). The median pain score of the moderate–severe depression group (3 [2, 3]) was also higher than that of the none‐mild depression group (2 [1, 2], *p* < 0.001). The median IES score was considerably higher in the moderate–severe group (33.0 [22.0–48.0)] than in the none‐mild group (22.0 [5.5–30.0], *p* < 0.001), while the mean FSI score was substantially lower in the none‐mild group (14 [7–21]) than in the moderate–severe group (34 [26–47], *p* < 0.001). Among patients with moderate–severe depression, 14 (32.6%) also experienced moderate–severe anxiety (Table 2).

**TABLE 2:** Influencing factors of depression and anxiety in cancer patients who experienced serious adverse events.

Variables	PHQ‐9 (*N* = 112)		*p* Value	GAD‐7 (*N* = 112)		*p* Value
	None‐mild (*n* = 69)	Moderate–severe (*n* = 43)		None‐mild (*n* = 97)	Moderate–severe (*n* = 15)	
Sex			0.174			0.405
Male	38 (55.1)	18 (41.9)		50 (51.5)	6 (40.0)	
Female	31 (44.9)	25 (58.1)		47 (48.5)	9 (60.0)	
Age (years), median (inter‐quartile range [IQR])	60.0 (56.0–66.5)	59.0 (50.0–68.0)	0.369	59.0 (56.0–66.0)	57.0 (45.0–69.0)	0.295
Marital status			0.627			0.957
Married	67 (97.1)	41 (95.3)		93 (95.9)	15 (100.0)	
Unmarried	2 (2.9)	2 (4.7)		4 (4.1)	0 (0)	
Occupation			0.132			0.005
Full‐time	10 (14.5)	3 (7.0)		11 (11.3)	2 (13.3)	
Part‐time	0 (0)	3 (7.0)		0 (0)	3 (20.0)	
Unemployed	31 (44.9)	22 (51.2)		46 (47.6)	7 (46.7)	
Retired	24 (34.8)	14 (32.6)		35 (36.1)	3 (20.0)	
Others	4 (5.8)	1 (2.3)		5 (5.2)	0 (0)	
Education level			0.731			0.338
High school level or below	56 (81.2)	36 (83.7)		81 (83.5)	11 (73.3)	
College/university and above	13 (18.8)	7 (16.3)		16 (16.5)	4 (26.7)	
Living alone			0.503			0.736
Yes	13 (18.8)	6 (14.0)		16 (16.5)	3 (20.0)	
No	56 (81.2)	37 (86.0)		81 (83.5)	12 (80.0)	
Frequency of acquiring medical information			0.020			0.690
Frequently (several times per month)	50 (72.5)	39 (90.7)		76 (78.4)	13 (86.7)	
Occasionally (several times per year)	19 (27.5)	4 (9.3)		21 (21.6)	2 (13.3)	
First treatment			0.019			0.100
Yes	33 (47.8)	11 (25.6)		41 (42.3)	3 (20.0)	
No	36 (52.2)	32 (74.4)		56 (57.7)	12 (80.0)	
Participating in clinical trials for the first time			0.957			0.939
Yes	64 (92.8)	40 (93.0)		90 (92.8)	14 (93.3)	
No	5 (7.2)	3 (7.0)		7 (7.2)	1 (6.7)	
Stage of clinical trial			0.281			0.558
I	11 (15.9)	4 (9.3)		14 (14.4)	1 (6.7)	
II	15 (21.7)	6 (14.0)		17 (17.5)	4 (26.7)	
III	43 (62.3)	33 (76.7)		66 (68.0)	10 (66.7)	
Type of cancer			0.465			0.890
Lung	15 (21.7)	8 (18.6)		20 (20.6)	3 (20.0)	
Breast	16 (23.2)	9 (20.9)		21 (21.6)	4 (26.7)	
Liver	13 (18.8)	6 (14.0)		18 (18.6)	1 (6.7)	
Gastric	8 (11.6)	10 (23.3)		14 (14.4)	4 (26.7)	
Esophagus	2 (2.9)	1 (2.3)		3 (3.1)	0 (0)	
Cervical	2 (2.9)	2 (4.7)		3 (3.1)	1 (6.7)	
Ovarian	1 (1.4)	2 (4.7)		3 (3.1)	0 (0)	
Prostate	3 (4.3)	0 (0)		3 (3.1)	0 (0)	
Hematologic	3 (4.3)	4 (9.3)		6 (6.2)	1 (6.7)	
Other	6 (8.7)	1 (2.3)		6 (6.2)	1 (6.7)	
Pain score, median (IQR)	2 (1–2)	3 (2–3)	0.000	2 (0–2)	3 (2–4)	0.000
Charlson Comorbidity Index, median (IQR)	0 (0–2.5)	1 (0–3.0)	0.144	1 (0–2.0)	1 (0–6.0)	0.636
Impact of Events Scale, median (IQR)	22.0 (5.5–30.0)	33.0 (22.0–48.0)	0.000	22.0 (12.0–31.0)	48.0 (39.0–60.0)	0.000
Fatigue symptom inventory, median (IQR)	14.0 (7.0–21.0)	34.0 (26.0–47.0)	0.000	18.0 (9.5–28.0)	47.0 (34.0–53.0)	0.000
Life events, median (IQR)	0 (0–1)	1 (0–1)	0.305	0 (0–1)	1 (0–2)	0.025
Physical function (EORTC QLQ‐C30), median (IQR)	93.3 (80.0–100.0)	66.7 (60.0–80.0)	0.000	86.7 (73.3–100.0)	60.0 (33.3–73.3)	0.000
Role function (EORTC QLQ‐C30), median (IQR)	100.0 (100.0–100.0)	66.7 (50.0–100.0)	0.000	100.0 (83.3–100.0)	66.7 (33.3–66.7)	0.000
Emotional function (EORTC QLQ‐C30), median (IQR)	100.0 (83.3–100.0)	66.7 (66.7–75.0)	0.000	91.7 (70.8–100.0)	58.3 (33.3–66.7)	0.000
Cognitive function (EORTC QLQ‐C30), median (IQR)	100.0 (83.3–100.0)	66.7 (50.0–83.3)	0.000	83.3 (66.7–100.0)	50.0 (33.3–83.3)	0.000
Social function (EORTC QLQ‐C30), median (IQR)	100.0 (66.7–100.0)	66.7 (50.0–66.7)	0.000	83.3 (66.7–100.0)	50.0 (33.3–66.7)	0.000
Global quality of life (EORTC QLQ‐C30), median (IQR)	66.7 (66.7–83.3)	50.0 (41.7–66.7)	0.000	66.7 (58.3–83.3)	50.0 (33.3–50.0)	0.000
PHQ‐9						0.000
None/mild				68 (70.1)	1 (6.7)	
Moderate to severe				29 (29.9)	14 (93.3)	
GAD‐7			0.000			
None/mild	68 (98.6)	29 (67.4)				
Moderate to severe	1 (1.4)	14 (32.6)				

In total, 97 participants (86.6%) had none‐mild anxiety and 15 (13.4%) had moderate–severe anxiety. The median GAD‐7 score was 2 (0–6.5) and 14 (11–14), respectively. Patients in the moderate–severe anxiety group had a higher median pain score (3 [2–4] than those in the none‐mild anxiety group (2 (0–2), The median IES score in the moderate–severe anxiety group (48 [39–60]) was significantly higher than that in the none‐mild anxiety group (22 [12–31]), as was the FSI score: 47 (34–53) versus 18 (9.5–28). In the moderate–severe anxiety group, 14 (93.3%) patients also had moderate–severe depression (Table 2).

Table 3 shows the results of the logistic regression analysis of depression and anxiety in cancer patients who experienced SAEs. Logistics regression analysis revealed that participants who acquired medical information occasionally were more depressed than those who acquired medical information more frequently (OR 28.792, 95% CI 1.610–514.991, *p* = 0.022). Lower cognitive function (OR 0.907, 95% CI 0.824–0.998, *p* = 0.046) and lower global quality of life (OR 0.945, 95% CI 0.898–0.995, *p* = 0.031) were associated with depression in cancer patients who had experienced SAEs. Pain (OR 31.515, 95% CI 1.055–941.124, *p* = 0.046), low emotional function (OR 0.725, 95% CI 0.540–0.973, *p* = 0.032), low global quality of life (OR 0.893, 95% CI 0.812–0.983, *p* = 0.020), and a high IES score (OR 1.258, 95% CI 1.042–1.519, *p* 0.029) were associated with anxiety in cancer patients with SAEs (Table 3).

**TABLE 3:** Logistic regression analysis of depression and anxiety in cancer patients who experienced SAEs.

Variables	OR	95% CI	*p*
Depression			
Frequency of acquiring medical information			
Frequently (several times per month)	1.000		
Occasionally (several times per year)	28.792	1.610–514.991	0.022
Cognitive function (EORTC QLQ‐C30)	0.907	0.824–0.998	0.046
Global quality of life (EORTC QLQ‐C30)	0.945	0.898–0.995	0.031
Anxiety			
Pain	31.515	1.055–941.124	0.046
Emotional function (EORTC QLQ‐C30)	0.725	0.540–0.973	0.032
Global quality of life (EORTC QLQ‐C30)	0.893	0.812–0.983	0.020
Impact of Events Scale	1.258	1.042–1.519	0.029

**FIGURE 1:**
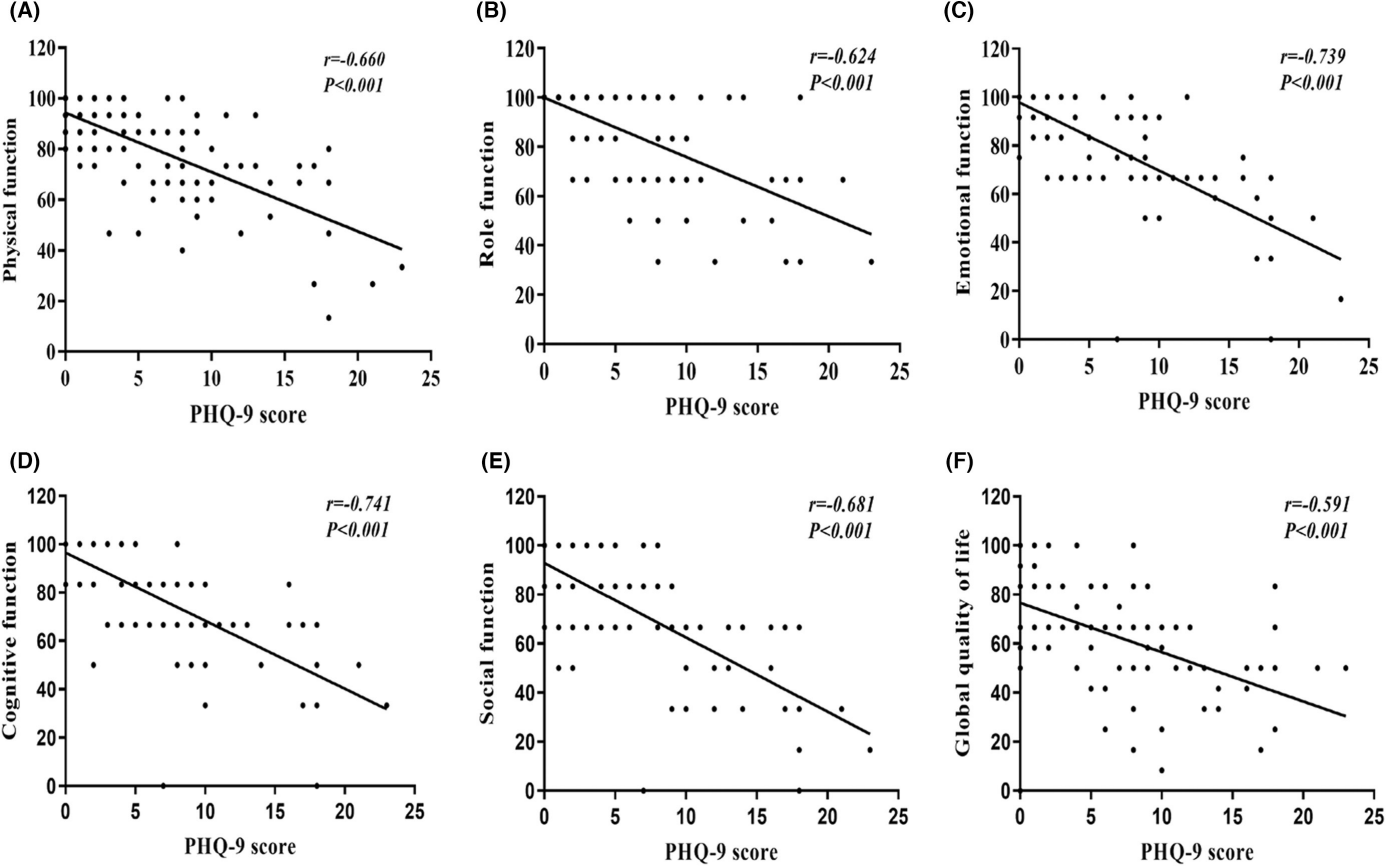
Correlation of depression with quality of life (A) PHQ‐9 with physical function (B) PHQ‐9 with role function (C) PHQ‐9 with emotional function (D) PHQ‐9 with cognitive function (E) PHQ‐9 with social function (F) PHQ‐9 with global quality of life.

Quality of life was significantly better in the none‐mild than in the moderate–severe depression group. The median physical function (93.3 [80.0–100.0]), role function (100.0 [100.0–100.0]), emotional function (100.0 [83.3–100.0]), cognitive function (100.0 [83.3–100.0]), social function (100.0 [66.7–100.0]), and global quality of life (66.7 [66.7–83.3]) scores were significantly higher in the none‐mild than the moderate–severe depression group (*p* < 0.001; Table 2). The PHQ‐9 score correlated negatively with the physical function score (*r* = −0.660, *p* < 0.001), role function score (*r* = −0.624, *p* < 0.001), emotional function score (*r* = −0.739, *p* < 0.001), cognitive function score (*r* = −0.741, *p* < 0.001), social function score (*r* = −0.681, *p* < 0.001), and global quality of life score (*r* = −0.591, *p* < 0.001; Figure 1).

**FIGURE 2:**
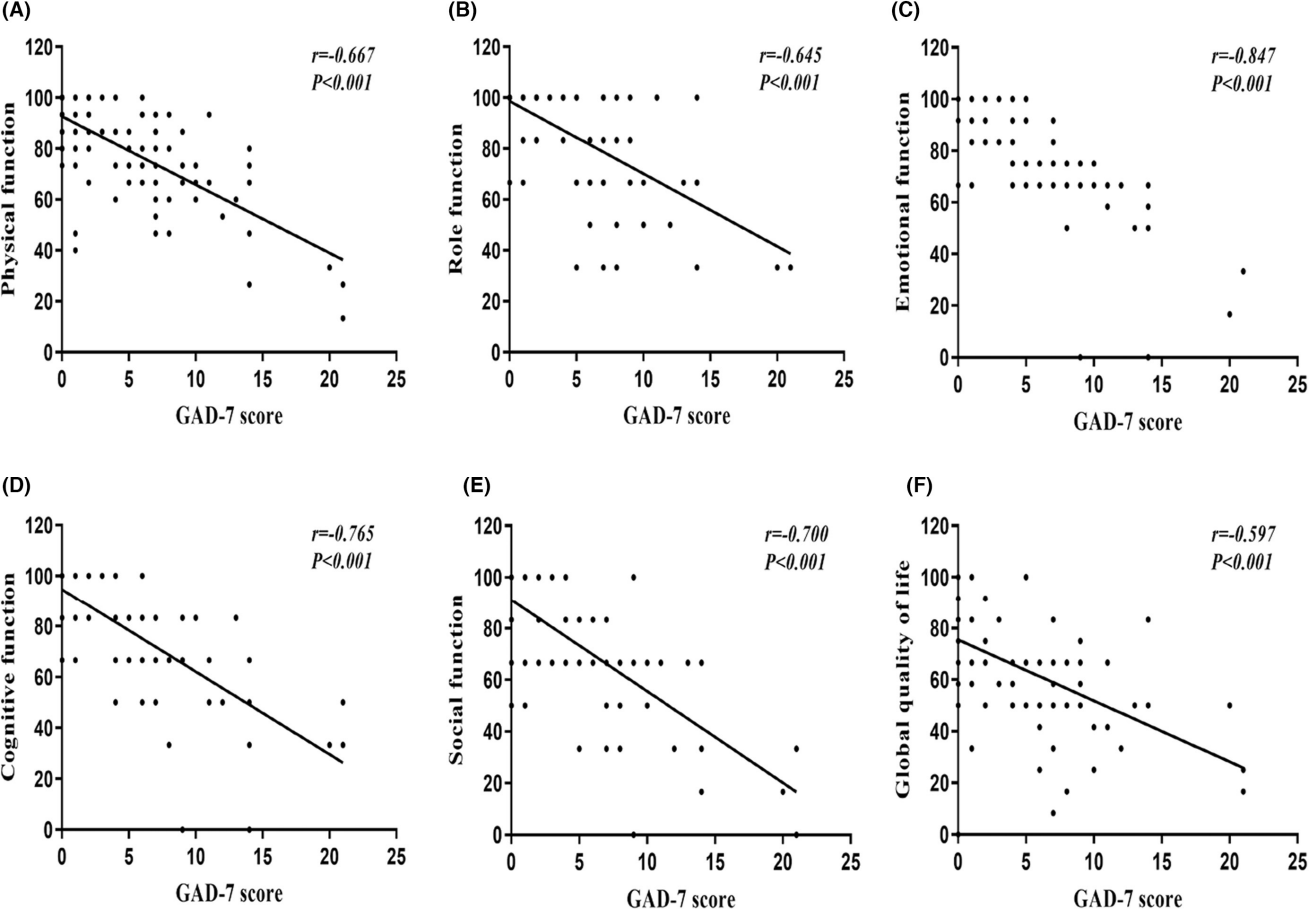
Correlation of anxiety with quality of life (A) GAD‐7 with physical function (B) GAD‐7 with role function (C) GAD‐7 with emotional function (D) GAD‐7 with cognitive function (E) GAD‐7 with social function (F) GAD‐7 with global quality of life.

The median physical function (86.7 [73.3–100.0]), role function (100.0 [83.3–100.0]), emotional function (91.7 [70.8–100.0]), cognitive function (83.3 [66.7–100.0]), social function (83.3 [66.7–100.0]), and global quality of life (66.7 [58.3–83.3]) scores were significantly higher in the none‐mild than in the moderate–severe anxiety group (*p* < 0.001; Table 2). The GAD‐7 score correlated negatively with the scores for physical function (*r* = −0.667, *p* < 0.001), role function (*r* = −0.645, *p* < 0.001), emotional function (*r* = −0.847, *p* < 0.001), cognitive function (*r* = −0.765, *p* < 0.001), social function (*r* = −0.700, *p* < 0.001), and global quality of life (*r* = −0.597, *p* < 0.001; Figure 2).

## 4 DISCUSSION

This study investigated anxiety and depression in 112 cancer patients enrolled in oncology clinical trials who experienced SAEs and who were assessed for anxiety and depression. Moderate–severe anxiety and depression were detected in 13.4% and 38.4% of the patients, respectively. Cancer patients who had suffered SAEs had a significantly higher risk of depression than those in earlier trials (15%–29%).^12^ Quality of life and cancer‐specific stress were risk factors for anxiety and depression. We also found that 32.6% of the patients in the moderate–severe depression group had moderate–severe anxiety, while 93.3% of those in the moderate–severe anxiety group had moderate–severe depression. These results indicate that anxiety and depression often coexist in cancer patients who have experienced SAEs, particularly those with anxiety. Therefore, it is necessary to screen cancer patients with SAEs for anxiety and depression so that these disorders can be detected promptly.

Psychological evaluation is not a routine part of most oncology clinical trials in China. Our findings demonstrate the need to assess anxiety and depression in clinical trials, especially in cancer patients who have had SAEs. The risk variables determined in this study may aid clinicians in identifying patients who need closer monitoring. Previous studies of cancer patients have shown that education level is related to depression, but that was not the case in this study.^33^, ^34^, ^35^ Rather, the frequency of access to medical information was an important risk factor for depression; in our patients those with occasional access to medical information had significantly higher levels of depression than those with frequent access. The probable reason for this was that a lack of medical information, particularly among those who were participating in a clinical trial for the first time, imposed a greater psychological burden after an SAE occurred.^36^ Logistic regression analysis revealed that cancer patients who acquired medical information occasionally were significantly more depressed than those who acquired it more frequently, suggesting that the frequency of acquisition of medical information may be an important predictor of depression. Understanding medical information plays an important role in reducing the likelihood of depression in cancer patients who have experienced SAEs. Our findings suggest the need to educate cancer patients about clinical trials, particularly with respect to drug‐induced adverse events.

This study also revealed that cancer patients with SAEs who did not receive therapy for the first time exhibited considerably higher levels of depression than those undergoing first‐time treatment, probably as a result of the ineffectiveness of prior therapies. Previous research has demonstrated that anxiety and depression are more common in cancer patients who work full‐time, and that both conditions may be made worse by work‐related stress.^30^ However, in this study the cancer patients who experienced SAEs and had moderate–severe anxiety were unemployed, such that their anxiety may have been due to their low knowledge level and lack of stable income.^37^ A previous study reported that the prevalence of fatigue in cancer patients was 15%.^38^ Similar to previous studies, we found that fatigue was significantly correlated with anxiety and depression.^39^ It is necessary to pay more attention to cancer patients with fatigue, especially those who have experienced SAEs. However, fatigue is a symptom of both cancer and depression, where this overlap may have a confounding effect; further research is needed.^1^

Similar to previous studies, pain was associated with anxiety and depression in the cancer patients who had experienced SAEs in this study.^40^ Previous studies have shown that pain can affect the quality of life of cancer patients.^41^ Cancer patients who experience SAEs and pain often exhibit anxiety and depression. The logistic regression analysis also suggested that pain is an important predictor of anxiety. Therefore, it is necessary to consider pain when screening cancer patients; early intervention for those with high pain scores can help reduce anxiety and depression.

Poor quality of life can cause various psychiatric disorders.^41^ Anxiety and depression in our cancer patients who had experienced SAEs were related to quality of life. The physical function, role function, emotional function, cognitive function, social function, and global quality of life EORTC QLQ‐C30 sub‐scale scores of the none‐mild depression/anxiety group were significantly better than those of the moderate–severe depression/anxiety group, suggesting that quality of life had a greater impact on anxiety and depression in cancer patients who had experienced SAEs. In particular, the Cognitive function score was an important predictor of depression in cancer patients who had experienced SAEs. Clinicians should pay attention to indicators of emotional distress as well as the symptoms of cancer.^30^ Higher quality of life is not only conducive to patient recovery but also reduces the psychological burden on patients. Anxiety and depression were negatively correlated with the physical function, role function, emotional function, cognitive function, social function, and global quality of life scores in this study. The quality of life score is useful to screen for anxiety and depression in cancer patients who have experienced SAEs.

The ASCO guidelines state that in screening cancer patients for anxiety and depression, sex, other chronic medical conditions, and marital status should be taken into account.^16^ However, these factors were not associated with either condition in our patients. Additionally, despite previous research findings to the contrary,^42^ there was no evidence of an increase in anxiety or depression in older patients.

More than 50% of patients with hematological cancers and gastric cancer had moderate–severe depression, and the incidence of depression in those with lung cancer was higher than in previous studies.^38^ Patients with gastric cancer and cervical cancer also had high rates of anxiety. The rate of moderate–severe anxiety in patients with gastric cancer was 22.2%, which was higher than in previous studies (17.7%).^43^ However, further research is needed due to the possible influence of different characteristics and treatment methods among cancer types.^44^

This study also had several limitations. Due to its cross‐sectional nature, the causal relationship between SAE and anxiety/depression could not be determined; prospective studies with appropriate control groups are therefore needed. Additionally, there was no follow‐up of the patients, so changes in anxiety and depression were not tracked. According to some studies, continuous monitoring of anxiety and depression is important for accurate assessment thereof.^35^ Therefore, it is necessary to study changes in anxiety and depression in cancer patients who have experienced SAEs. Also, although the ASCO guidelines recommend screening at diagnosis and treatment initiation, we did not establish the time since diagnosis in our patients, which may have affected the results and model predictions.^16^ The small sample size of this study was an additional limitation that should be addressed in follow‐up studies. Due to the paucity of data, risk factors such as progressing or progressive disease, substance use/abuse, and insomnia were not evaluated.^12^ Patients who required hospitalization and were in a critical condition were not included in this study, but the risk of a significant psychological burden in this population should be examined. Due to the limited sample sizes in some cancer subgroups and to account for the influence of ethnicity, region, illness features, and treatments, additional, systematic studies should be conducted. Moreover, it should be noted that, while the focus of this study was on cancer patients participating in clinical trials who had SAEs, SAEs can also occur in other patients and should accordingly also be investigated.

## 5 CONCLUSION

Cancer patients enrolled in clinical trials are at particular risk of drug‐induced side effects, and thus SAEs. This study examined the anxiety and depression levels of cancer patients who experienced SAEs while participating in oncology clinical trials. The results showed that cancer patients who had experienced SAEs had high rates of anxiety and depression, particularly the latter. The prevalence of moderate–severe depression was noticeably greater than previously reported. Poor quality of life, cancer‐specific stress and pain were associated with depression and anxiety symptoms in patients who had experienced SAEs. It is possible that the risk of anxiety/depression among the patients was due to their expectations about or experiences with the clinical trial, suggesting the need to evaluate anxiety and depression, and education about clinical trials among cancer patients with SAEs.

## AUTHOR CONTRIBUTIONS

**Zhen Peng:** Conceptualization (lead); formal analysis (lead); investigation (lead); resources (equal); writing – original draft (lead); writing – review and editing (equal). **Chongwei Wang:** Data curation (equal); investigation (lead); project administration (supporting); resources (equal); writing – original draft (equal); writing – review and editing (equal). **Yubei Sun:** Formal analysis (equal); investigation (equal); resources (lead); supervision (equal); writing – original draft (equal); writing – review and editing (equal). **Yan Ma:** Formal analysis (supporting); investigation (equal); project administration (supporting); resources (equal); writing – original draft (equal); writing – review and editing (equal). **Jumei Wang:** Formal analysis (supporting); investigation (equal); project administration (supporting); resources (supporting); writing – original draft (equal); writing – review and editing (equal). **Fei Xu:** Formal analysis (supporting); investigation (equal); project administration (supporting); resources (equal); writing – original draft (equal); writing – review and editing (equal). **Xiaoling Xu:** Investigation (supporting); project administration (supporting); resources (equal); writing – original draft (equal); writing – review and editing (equal). **Yin Chen:** Conceptualization (lead); formal analysis (equal); investigation (equal); methodology (lead); project administration (lead); resources (equal); supervision (equal); writing – original draft (lead); writing – review and editing (equal).

## CONFLICT OF INTEREST STATEMENT

The authors declare that they have not competing interests.

## ETHICS STATEMENT

This study was performed in line with the principles of the Declaration of Helsinki. Approval was granted by the Medical Research Ethics Committee of the First Affiliated Hospital of University of Science and Technology of China (approval no. 2021266).

## Data Availability

The data that support the findings of this study are available from the corresponding author upon reasonable request.
