# Correction to “Depression and anxiety in cancer patient enrolled in clinical trials with serious adverse events”

**DOI:** 10.1002/cam4.7426

**Published:** 2024-06-25

Peng Z, Wang C, Sun Y, et al. Depression and anxiety in cancer patient enrolled in clinical trials with serious adverse events. Cancer Med. 2023;12(19):20015‐20026. doi:10.1002/cam4.6556

In the “Author affiliation” section, the text

“^1^Division of Life Sciences and Medicine, Drug Clinical Trial Institution, The First Affiliated Hospital of USTC, University of Science and Technology of China, Anhui, Hefei, China

^2^Division of Life Sciences and Medicine, Department of Oncology, The First Affiliated Hospital of USTC, University of Science and Technology of China, Hefei, Anhui, China

^3^Division of Life Sciences and Medicine, Department of Rheumatology and Immunology, The First Affiliated Hospital of USTC, University of Science and Technology of China, Hefei, Anhui, China

^4^Division of Life Sciences and Medicine, Department of Endocrinology, The First Affiliated Hospital of USTC, University of Science and Technology of China, Hefei, Anhui, China

^5^Division of Life Sciences and Medicine, Department of Pulmonary and Critical Care Medicine, The First Affiliated Hospital of USTC, University of Science and Technology of China, Hefei, Anhui, China

^6^Division of Life Sciences and Medicine, Department of Scientific Research, The First Affiliated Hospital of USTC, University of Science and Technology of China, Hefei, Anhui, China” was incorrect.

**This should be**:

“^1^Drug Clinical Trial Institution, The First Affiliated Hospital of USTC, Division of Life Sciences and Medicine, University of Science and Technology of China, Hefei, Anhui, China.

^2^Department of Oncology, The First Affiliated Hospital of USTC, Division of Life Sciences and Medicine, University of Science and Technology of China, Hefei, Anhui, China

^3^Department of Rheumatology and Immunology, The First Affiliated Hospital of USTC, Division of Life Sciences and Medicine, University of Science and Technology of China, Hefei, Anhui, China

^4^Department of Endocrinology, The First Affiliated Hospital of USTC, Division of Life Sciences and Medicine, University of Science and Technology of China, Hefei, Anhui, China

^5^Department of Pulmonary and Critical Care Medicine, The First Affiliated Hospital of USTC, Division of Life Sciences and Medicine, University of Science and Technology of China, Hefei, Anhui, China

^6^Department of Scientific Research, The First Affiliated Hospital of USTC, Division of Life Sciences and Medicine, University of Science and Technology of China, Hefei, Anhui, China.”

In the “Correspondence” section, the text “Division of Life Sciences and Medicine, Department of Scientific Research, The First Affiliated Hospital of USTC, University of Science and Technology of China, Hefei, Anhui, China” was incorrect. **This should be**: “Department of Scientific Research, The First Affiliated Hospital of USTC, Division of Life Sciences and Medicine, University of Science and Technology of China, Hefei, Anhui, China.”

We apologize for this error.

